# Virtual State Coupled Sliding Mode Control: An Energy Exchange Approach with Tunable Performance Trade-Off

**DOI:** 10.3390/s26113381

**Published:** 2026-05-26

**Authors:** Jialong Wang, Jianli Wang, Jiaxin Jing, Canyang Zhao, Lei Zhang

**Affiliations:** College of Electrical Engineering, Henan University of Technology, Zhengzhou 450001, China; wangjialong@stu.haut.edu.cn (J.W.); jianli@stu.haut.edu.cn (J.W.); 241080400127@stu.haut.edu.cn (J.J.); 241080200525@stu.haut.edu.cn (C.Z.)

**Keywords:** sliding mode control, virtual state coupling, energy exchange, bilinear product coupling, transient performance trade-off

## Abstract

Traditional sliding mode control (SMC) lacks an active mechanism for redistributing energy among state channels during transient convergence, resulting in a rigid trade-off between response speed, overshoot suppression, and energy efficiency. This paper proposes a virtual state coupled SMC method that introduces a dynamic virtual state with bilinear product coupling $x_{1}x_{2}$ into the sliding surface. Unlike conventional virtual states that serve as static linear combinations or observer-based estimates, the proposed virtual state evolves dynamically and establishes an active energy exchange channel between the real and virtual state dynamics. Linearization and Lyapunov-based analyses prove local asymptotic stability of the closed-loop system. The coupling strength γ is shown to be decoupled from the linearized local eigenvalues and thus governs the energy–performance trade-off independently, while the condition c>γ/4 guarantees a non-vanishing domain of attraction. Simulations demonstrate that the proposed method achieves up to 53.2% control energy reduction under disturbance-free conditions compared with conventional SMC. Under persistent high-frequency disturbances, increasing γ reduces oscillations by 54.2% at a controllable energy cost of 45.7%. Systematic parameter selection guidelines are provided, and Monte Carlo simulations (500 trials, ±30% parameter perturbations) confirm 100% convergence. The proposed method offers an independently adjustable energy–performance trade-off mechanism suitable for sensor-based motion systems with stringent transient and energy requirements.

## 1. Introduction

As a classic nonlinear robust control method, SMC has been widely applied in strongly nonlinear and disturbance-prone engineering fields, such as motor drives, aircraft attitude control, and robot servo systems, owing to its full insensitivity to matched disturbances and fast response characteristics [1,2]. Once the system states reach the predefined sliding surface, the closed-loop dynamics are uniquely determined by the sliding surface, endowing the system with inherent robustness against external disturbances and parameter perturbations, making it one of the mainstream solutions for uncertain system control [3,4]. However, traditional linear sliding mode control has two inherent limitations that are difficult to reconcile, restricting its applicability in high-precision and low-energy-consumption scenarios: the first is the chattering problem. The discontinuous switching term in the control law induces high-frequency oscillation of the output, which not only excites unmodeled dynamics but also accelerates the mechanical wear of actuators and reduces the service life of the system [5,6]; the second is the rigid constraint of transient response. The traditional linear sliding surface can only achieve exponential convergence of states, and the convergence rate is uniquely determined by the coefficients of the sliding surface. This makes it difficult to flexibly trade off among convergence speed, overshoot suppression, and control energy consumption. Moreover, it cannot actively adjust the energy distribution during the convergence process, limiting the optimization space for transient performance [7,8].

The root cause of the above limitations is that traditional sliding mode control lacks an active energy redistribution mechanism during the convergence process. Interestingly, a structurally analogous phenomenon appears in classical rigid body dynamics: the tennis racket theorem (TRT) indicates that the motion of a rigid body is stable when rotating around the maximum or minimum principal axis of inertia, while when rotating around the intermediate principal axis of inertia, a tiny initial disturbance can trigger periodic energy exchange of angular velocity among the three principal axes of inertia, thereby inducing the tumbling phenomenon [9,10]. Its mathematical essence is that the product coupling term between state variables can drive the active redistribution of energy among state channels [11]. While the physical context differs fundamentally from controlled transient response, the underlying mathematical structure—bilinear state-product coupling driving energy redistribution among dynamic channels—suggests a design principle that has not previously been exploited in SMC: synthetically introducing product coupling into the controller dynamics to realize controllable energy flow during convergence. We emphasize that the TRT serves as a conceptual inspiration for the product-coupling structure, not as a direct physical correspondence.

To address the inherent limitations of traditional SMC, extensive research has been conducted in academia: In terms of chattering suppression, the boundary layer method [12] suppresses chattering by constructing a smooth boundary layer near the switching function and replacing the discontinuous sign function with a saturation function, but at the cost of sacrificing steady-state accuracy. High-order sliding mode control [13,14] applies the discontinuous control action to the high-order derivative of the sliding variable, which can suppress chattering while retaining robustness. However, it suffers from a complex controller structure, strict requirements for state observation accuracy, and high difficulty in engineering implementation. In terms of transient response optimization, terminal sliding mode control [15,16] achieves finite-time convergence of states by constructing a nonlinear sliding surface, but its convergence time is significantly affected by the initial state, and no general parameter-tuning criterion exists. Integral sliding mode control [17,18] eliminates steady-state errors by introducing an integral term, but it is prone to integral windup, leading to increased overshoot and degraded transient performance. Adaptive sliding mode control [19,20] copes with system uncertainties by online tuning of the switching gain, but its design goal focuses exclusively on robustness improvement and cannot achieve active regulation of transient response [21]. In addition, most existing research on virtual states embeds them into the sliding surface to achieve state decoupling or controller order reduction and simplification. However, such virtual states are static linear combinations, and they do not introduce the dynamic coupling required for energy exchange [22,23]. Energy-shaping methods, such as the controlled Lagrangian framework [24] and Interconnection and Damping Assignment Passivity-Based Control (IDA-PBC) [25], can achieve energy redistribution through state coupling, but they require accurate model solutions of partial differential equations and cannot inherit the inherent robustness of sliding mode control. Several emerging approaches, such as state-filtered disturbance rejection control [26] and multilayer neuroadaptive reinforcement learning via actor–critic mechanisms [27], provide powerful alternatives for disturbance estimation and adaptive optimization, yet they focus on disturbance rejection and learning rather than on active energy redistribution for transient shaping. Several recent studies have exploited the inherent coupling of specific systems to improve energy efficiency [28,29,30], but they rely on the existing local coupling structure of the system, provide no independent coupling strength adjustment parameter, and offer limited generality [31,32,33,34]. In the broader SMC literature, methods such as active unmatched disturbance rejection quasi-sliding observers [35], robust adaptive barrier function-based SMC [36], and adaptive integral SMC for steer-by-wire systems [37] have been proposed, yet none provides a mechanism for constructing synthetic, independently tunable energy exchange channels within the SMC framework.

To address this issue, this paper proposes a novel sliding mode control method based on virtual state coupling and an energy exchange mechanism. At the controller design level, by introducing a dynamic virtual state with a product coupling term and integrating it into the sliding surface, a coupled dynamic system between the real state and the virtual state is constructed, which achieves active regulation and redistribution of energy among state channels.

The main contributions of this paper are summarized as follows:

(1) Virtual state coupling mechanism: A dynamic virtual state with bilinear product coupling $\dot{z}=x_{1}x_{2}-\rho z$ is embedded into the sliding surface, establishing—to the best of our knowledge, for the first time in SMC—an active energy exchange channel whose coupling strength γ serves as an independent tuning parameter.
(2) Stability and decoupling analysis: Lyapunov-based analysis proves local asymptotic stability and explicitly characterizes the domain of attraction through the condition c>γ/4. The coupling strength γ is shown to be decoupled from the linearized eigenvalues while affecting the domain of attraction, providing an independent dimension for transient performance optimization.
(3) Quantified energy–performance trade-off: Systematic simulations demonstrate up to 53.2% control energy reduction (disturbance-free) and a tunable trade-off between response smoothness (54.2% oscillation reduction) and energy cost (45.7% increase) under persistent disturbances.
(4) Practical design guidelines: A step-by-step parameter selection protocol is provided, analytically characterizing the influence of key parameters (c,γ,ρ,k) on settling time, control energy, and chattering level.

Based on the above research objectives, the rest of this paper is organized as follows: Section 2 formulates the problem; Section 3 introduces the controller design; Section 4 conducts stability analysis; Section 5 verifies the effectiveness of the proposed method through simulations; Section 6 concludes the whole paper.

Recent sliding mode control developments reported in the *Sensors* journal include observer-based compensation [38], optimal fault-tolerant control with explicit energy-performance trade-offs [39], and piecewise sliding mode-enhanced active disturbance rejection control [40].

## 2. Problem Formulation

Consider a typical second-order integrator system [41]:

(1) $$\left\{\begin{array}{c} {\dot{x}}_{1}=x_{2}\\ {\dot{x}}_{2}=u+d\left(t\right) \end{array}\right.$$

where $x_{1}$ is the position error (with the target of 0), $x_{2}$ is the velocity, *u* is the control input, and d(t) is the external disturbance. The conventional sliding mode controller usually selects a linear sliding surface $\sigma =x_{2}+cx_{1}$ (c>0) and adopts the constant rate reaching law $\dot{\sigma }=-k\mathrm{sign}\left(\sigma \right)$ to derive the control law $u=-cx_{2}-k\mathrm{sign}\left(\sigma \right)$ [42]. When the system enters the sliding mode σ=0, the reduced-order system is ${\dot{x}}_{1}=-cx_{1}$, realizing exponential convergence. However, the resulting dynamics are linear with fixed parameters, preventing regulation of energy distribution during the convergence process and making the system prone to overshoot or excessive energy consumption [43].

**Remark 1**  (Scope and limitations)**.** *System (1) represents the canonical second-order integrator with matched disturbance—the foundational benchmark for SMC design [41]. While this formulation assumes matched uncertainty, it serves as the necessary starting point for establishing the virtual state coupling framework. The extension to systems with mismatched uncertainties and higher-order dynamics is discussed in Section 6 as future work.*

## 3. Design of Sliding Mode Controller Based on Virtual State Coupling

To overcome these limitations, this paper draws inspiration from the tennis racket theorem. This theorem indicates that the rotation of a rigid body around the maximum or minimum principal axis of inertia is stable, while when rotating around the intermediate axis, a tiny disturbance will cause the angular velocity to exchange periodically among the axes, triggering the flip phenomenon. Its mathematical essence is that the product coupling $\omega_{1}\omega_{2}$ between state variables drives the redistribution of energy, forming an energy exchange mechanism. If a similar structure can be introduced into the design of the sliding mode controller to actively regulate the distribution of energy among different state channels during the system’s convergence process, it is expected to optimize the transient response. Based on this, this paper intends to introduce a virtual state *z* and construct a product coupling term to simulate the energy exchange mechanism of the tennis racket effect, enabling the system to realize energy flow in the state space and optimize the transient response.

### 3.1. Introducing the Virtual State

We introduce the virtual state *z*:

(2) $$\dot{z}=x_{1}x_{2}-\rho z,\;\rho >0$$

Themotivation for this construction stems from the product coupling structure in the tennis racket theorem. The product term $x_{1}x_{2}$ emulates the cross-coupling effect of angular velocity in rigid body dynamics, which is the core mechanism driving the transfer of energy between different states. By introducing this nonlinear term, the virtual state can sense the interaction between the real states $x_{1}$ and $x_{2}$, thereby establishing an energy exchange channel in the system dynamics. The dissipation term −ρz prevents divergence of the virtual state that would otherwise result from the integral effect of the product term. The parameter ρ>0 controls the energy dissipation rate of the virtual state: the larger ρ is, the faster the energy of the virtual state decays, and the shorter the duration of the coupling effect. In the simulations, the initial condition is set as z(0)=0 unless otherwise specified, which means that the virtual state is initially inactive.

**Remark 2**  (Implementation considerations)**.** *In a digital implementation, the virtual state z is an internal variable of the controller, updated at the same sampling rate $T_{s}$ as the control loop via forward Euler discretization: $z_{k+1}=z_{k}+T_{s}\left(x_{1,k}x_{2,k}-\rho z_{k}\right)$. As long as $T_{s}\ll \min\left(1/c,1/\rho \right)$, the discretization error is negligible. At controller startup, z(0) may be initialized from the measured value of $x_{1}\left(0\right)$ by reading the position sensor before entering the control loop, which reduces the initial mismatch in practical implementation. Exact equality is not required; even if $z\left(0\right)\neq x_{1}\left(0\right)$ due to sensor delays or initialization errors, stability is preserved—only the transient trajectory is slightly affected. This is verified in Section 5.1.*

### 3.2. Sliding Surface Design

The virtual state is incorporated into the sliding surface, forming a coupled sliding surface:

(3) $$\sigma =x_{2}+cx_{1}+\gamma z,\;c>0,\;\gamma >0$$

This sliding surface adds a linear term γz of the virtual state *z* on the basis of the traditional linear combination $x_{2}+cx_{1}$. The parameter *c* controls the convergence rate of the traditional sliding surface, and γ is the coupling strength, which determines the influence degree of the virtual state on the sliding surface. When the system is in the sliding mode σ=0, it holds that $x_{2}=-cx_{1}-\gamma z$. After substituting the dynamics of the virtual state, a closed coupled system is formed between the real state $x_{1}$ and the virtual state *z*. The two can exchange energy through the product term $x_{1}x_{2}$. The value of the parameter γ directly affects the strength of this coupling: when γ>0, the virtual state contributes a term −γz that counteracts the state $x_{1}$ during convergence, helping to suppress overshoot. Since the local eigenvalues are independent of γ (see the analysis in Section 4), γ can be used as a free parameter to regulate the transient response characteristics.

### 3.3. Derivation of the Control Law

Taking the derivative of σ and substituting the system dynamics,

(4) $$\dot{\sigma }={\dot{x}}_{2}+c{\dot{x}}_{1}+\gamma \dot{z}=u+d\left(t\right)+cx_{2}+\gamma \left(x_{1}x_{2}-\rho z\right)$$

The constant rate reaching law $\dot{\sigma }=-k\mathrm{sign}\left(\sigma \right)$ is adopted. The disturbance is temporarily neglected to derive the equivalent control part, while the switching term −ksign(σ) is used to cope with the disturbance, and the control law is solved as follows:

(5) $$u=-cx_{2}-\gamma \left(x_{1}x_{2}-\rho z\right)-k\mathrm{sign}\left(\sigma \right)$$

Equation (5) comprises three components: the term $-cx_{2}$ represents the standard linear state feedback [41]; the term $-\gamma (x_{1}x_{2}-\rho z)$ is the virtual state coupling contribution—the core novelty of this work; and −ksign(σ) is the standard SMC switching term [1,41] for disturbance rejection. To alleviate chattering, the sign function can be replaced with the boundary-layer approximation sat(σ/δ) in practical applications, where δ>0 is the boundary layer thickness [12]. This boundary-layer saturation should not be confused with actuator amplitude saturation.

## 4. Stability Proof

This section conducts a rigorous analysis of the stability of the sliding mode system. First, the reduced-order dynamics of the system after it enters the sliding surface are derived. Then, the stability of the equilibrium point is proved via the linearization method and the Lyapunov method, respectively, and the influence law of the coupling strength γ on the stability is revealed.

### 4.1. Dynamics of the Sliding Phase

When the system enters the sliding surface σ=0, according to the definition of the sliding surface $\sigma =x_{2}+cx_{1}+\gamma z=0$, it holds that

(6) $$x_{2}=-cx_{1}-\gamma z$$

Substituting Equation (6) into the dynamics of the virtual state $\dot{z}=x_{1}x_{2}-\rho z$,

(7) $$\dot{z}=x_{1}\left(-cx_{1}-\gamma z\right)-\rho z=-cx_{1}^{2}-\gamma x_{1}z-\rho z$$

Meanwhile, from the system state equation ${\dot{x}}_{1}=x_{2}$ and Equation (6), it holds that

(8) $${\dot{x}}_{1}=-cx_{1}-\gamma z$$

Combining Equations (7) and (8), the complete dynamics equation of the sliding mode system is obtained:

(9) $$\left\{\begin{array}{c} {\dot{x}}_{1}=-c\;x_{1}-\gamma \;z\\ \dot{z}=-c\;x_{1}^{2}-\gamma \;x_{1}z-\rho \;z \end{array}\right.$$

This is a two-dimensional nonlinear autonomous system, and its equilibrium point satisfies ${\dot{x}}_{1}=0$ and $\dot{z}=0$. From ${\dot{x}}_{1}=0$, it holds that $-cx_{1}-\gamma z=0$, i.e., $z=-\frac{c}{\gamma }x_{1}$. Substituting this into $\dot{z}=0$,

(10) $$-cx_{1}^{2}-\gamma x_{1}\left(-\frac{c}{\gamma }x_{1}\right)-\rho \left(-\frac{c}{\gamma }x_{1}\right)=-cx_{1}^{2}+cx_{1}^{2}+\frac{\rho c}{\gamma }x_{1}=\frac{\rho c}{\gamma }x_{1}=0$$

Solving this yields $x_{1}=0$, and substituting back yields z=0. Therefore, the system (9) has a unique equilibrium point $\left(x_{1},z\right)=\left(0,0\right)$. Below, we establish that this equilibrium point is asymptotically stable.

### 4.2. Linearized Stability Analysis

First, the nonlinear system (9) is linearized at the equilibrium point (0,0). Let

(11) $$\left\{\begin{array}{c} f_{1}\left(x_{1},z\right)=-cx_{1}-\gamma z\\ f_{2}\left(x_{1},z\right)=-cx_{1}^{2}-\gamma x_{1}z-\rho z \end{array}\right.$$

Then, evaluating the Jacobian matrix $J=\left[\begin{array}{cc} \frac{\partial f_{1}}{\partial x_{1}} & \frac{\partial f_{1}}{\partial z}\\ \frac{\partial f_{2}}{\partial x_{1}} & \frac{\partial f_{2}}{\partial z} \end{array}\right]$ at the equilibrium point (0,0), we obtain $\frac{\partial f_{1}}{\partial x_{1}}=-c$, $\frac{\partial f_{1}}{\partial z}=-\gamma$; $\frac{\partial f_{2}}{\partial x_{1}}=-2cx_{1}-\gamma z$ evaluates to 0, and $\frac{\partial f_{2}}{\partial z}=-\gamma x_{1}-\rho$ evaluates to −ρ. Therefore, the Jacobian matrix at the equilibrium point is

(12) $$J=\left[\begin{array}{cc} -c & -\gamma \\ 0 & -\rho \end{array}\right]$$

The eigenvalues of this matrix are obtained from the characteristic equation det(λI−J)=0:

(13) $$\det\left[\begin{array}{cc} \lambda +c & \gamma \\ 0 & \lambda +\rho \end{array}\right]=\left(\lambda +c\right)\left(\lambda +\rho \right)=0$$

Solving this yields the eigenvalues $\lambda_{1}=-c$ and $\lambda_{2}=-\rho$. Since the controller parameters satisfy c>0 and ρ>0, both eigenvalues are negative real numbers. According to the linearization theorem (Lyapunov’s first method), when all the eigenvalues of the linearized system have negative real parts, the equilibrium point of the original nonlinear system is exponentially stable.

The expressions of the eigenvalues $\lambda_{1}=-c$ and $\lambda_{2}=-\rho$ do not contain the coupling strength γ. This establishes the following nuanced property: the linearized local exponential convergence rate near the origin is governed solely by *c* and ρ, and it is independent of γ. However, as will be shown in Section 4.3, the domain of attractiondepends on γ through the condition c>γ/4. Therefore, γ is decoupled from the local rate but coupled to the global stability region. This distinction is essential for practical design: γ can be freely tuned to shape transient response without affecting the local convergence speed, provided the condition c>γ/4 is satisfied.

### 4.3. Analysis of Nonlinear Stability Properties Based on Energy Function

The linearization analysis in Section 4.2 guarantees local exponential stability for any γ as long as c>0, ρ>0. However, it does not provide information about the size of the region of attraction, which is influenced by higher-order nonlinear terms such as $-cx_{1}^{2}$ and the coupling term $-\gamma x_{1}z$. To estimate the domain of attraction and to further reveal the energy exchange mechanism, we construct the following Lyapunov function candidate:

(14) $$E=\frac{1}{2}\left(x_{1}^{2}+\frac{\gamma }{\rho }z^{2}\right),\;\gamma >0,\rho >0$$

Motivation for the choice of *E*: To deeply reveal the coupling mechanism between the virtual state and the real state from the energy perspective, a positive definite function *E* is constructed whose time derivative can be naturally decomposed into the sum of a negative definite quadratic form and a higher-order disturbance term, thereby directly proving the asymptotic stability of the system. Analyzing the structure of the sliding mode system (9), ${\dot{x}}_{1}$ contains the linear dissipation term $-cx_{1}$ and the coupling term −γz; $\dot{z}$ contains the nonlinear term $-cx_{1}^{2}$ and the dissipation term −ρz. Inspired by the conversion principle of kinetic energy and potential energy in classical mechanics, the term $x_{1}^{2}$ is selected to characterize the “energy of the real state”, and the term $z^{2}$ is introduced to represent the “energy of the virtual state”. The coefficient γ/ρ is selected so that the dissipative contribution of the virtual state appears in a comparable quadratic-energy form, allowing the derivative of *E* to be decomposed into a dominant quadratic part and higher-order nonlinear terms.

The function *E* has the following properties:

1. E(0,0)=0.
2. When $\left(x_{1},z\right)\neq \left(0,0\right)$, E>0 (since γ>0, ρ>0 ensure the coefficients are positive).

Therefore, *E* is a positive definite function, and can be used as a candidate Lyapunov function. Then, take the time derivative of *E* along the system (9):

(15) $$\begin{array}{cc} \dot{E} & =\frac{\partial E}{\partial x_{1}}{\dot{x}}_{1}+\frac{\partial E}{\partial z}\dot{z}\\ \; & =x_{1}{\dot{x}}_{1}+\frac{\gamma }{\rho }z\dot{z}\\ \; & =x_{1}\left(-cx_{1}-\gamma z\right)+\frac{\gamma }{\rho }z\left(-cx_{1}^{2}-\gamma x_{1}z-\rho z\right) \end{array}$$

Expanding step by step, the first term gives $x_{1}\left(-cx_{1}-\gamma z\right)=-cx_{1}^{2}-\gamma x_{1}z$, and the second term gives $\frac{\gamma }{\rho }z\left(-cx_{1}^{2}-\gamma x_{1}z-\rho z\right)=-\frac{\gamma c}{\rho }x_{1}^{2}z-\frac{\gamma^{2}}{\rho }x_{1}z^{2}-\gamma z^{2}$. Adding the two terms together,

(16) $$\dot{E}=\left(-cx_{1}^{2}-\gamma x_{1}z\right)+\left(-\frac{\gamma c}{\rho }x_{1}^{2}z-\frac{\gamma^{2}}{\rho }x_{1}z^{2}-\gamma z^{2}\right)$$

After rearrangement, $\dot{E}$ is decomposed into the quadratic form part $Q\left(x_{1},z\right)$ and the higher-order small term part $H\left(x_{1},z\right)$:

(17) $$\dot{E}=-\underset{Q(x_{1},z)}{\underset{︸}{\left(cx_{1}^{2}+\gamma x_{1}z+\gamma z^{2}\right)}}-\underset{H(x_{1},z)}{\underset{︸}{\left(\frac{\gamma c}{\rho }x_{1}^{2}z+\frac{\gamma^{2}}{\rho }x_{1}z^{2}\right)}}$$

Analysis of the Quadratic Form Part $Q\left(x_{1},z\right)$ $Q\left(x_{1},z\right)=cx_{1}^{2}+\gamma x_{1}z+\gamma z^{2}$ can be expressed as the quadratic form $\left[\begin{array}{cc} x_{1} & z \end{array}\right]A\left[\begin{array}{c} x_{1}\\ z \end{array}\right]$, where the symmetric matrix *A* is

(18) $$A=\left[\begin{array}{cc} c & \gamma /2\\ \gamma /2 & \gamma \end{array}\right]$$

According to Sylvester’s criterion, the necessary and sufficient condition for a real symmetric matrix to be positive definite is that all its leading principal minors are greater than 0:

- First-order leading principal minor: c>0 (holds).
- Second-order leading principal minor: $\det\left(A\right)=c\cdot \gamma -{\left(\gamma /2\right)}^{2}=c\gamma -\frac{\gamma^{2}}{4}=\gamma \left(c-\frac{\gamma }{4}\right)$.

Therefore, the necessary and sufficient condition for $Q\left(x_{1},z\right)$ to be a positive definite quadratic form is

(19) $$\gamma \left(c-\frac{\gamma }{4}\right)>0$$

Since this paper considers γ>0 (positive coupling strength), this condition is simplified to

(20) $$c>\frac{\gamma }{4}$$

Condition (20) gives the relationship that the parameters *c* and γ need to satisfy. In the simulation of this paper, c=3 and γ=2 are taken, which obviously satisfies 3>2/4=0.5. When condition (20) holds, there exists a constant λ>0 (which can be taken as the minimum eigenvalue of *A*) such that

(21) $$Q\left(x_{1},z\right)\geq \lambda \left(x_{1}^{2}+z^{2}\right)$$

Analysis of the Higher-order Term Part $H\left(x_{1},z\right)$

(22) $$H\left(x_{1},z\right)=\frac{\gamma c}{\rho }x_{1}^{2}z+\frac{\gamma^{2}}{\rho }x_{1}z^{2}$$

is a cubic homogeneous polynomial of $\left(x_{1},z\right)$. Near the origin, there exists a constant M>0 such that

(23) $$\left|H\left(x_{1},z\right)\right|\leq M{\left(x_{1}^{2}+z^{2}\right)}^{3/2}$$

The establishment of this inequality is based on the following: when $\left(x_{1},z\right)$ is sufficiently small, ${\left(x_{1}^{2}+z^{2}\right)}^{3/2}$ is a small quantity of higher order than $\left(x_{1}^{2}+z^{2}\right)$, and both $\left|x_{1}^{2}z\right|$ and $\left|x_{1}z^{2}\right|$ can be bounded by ${\left(x_{1}^{2}+z^{2}\right)}^{3/2}$. Specifically, from the inequalities $\left|x_{1}\right|\leq \sqrt{x_{1}^{2}+z^{2}}$ and $\left|z\right|\leq \sqrt{x_{1}^{2}+z^{2}}$, we can obtain

(24) $$\left|x_{1}^{2}z\right|\leq {\left(x_{1}^{2}+z^{2}\right)}^{3/2},\;\left|x_{1}z^{2}\right|\leq {\left(x_{1}^{2}+z^{2}\right)}^{3/2}$$

Therefore, $M=\frac{\gamma c}{\rho }+\frac{\gamma^{2}}{\rho }$ can be taken.

Determination of the Local Neighborhood and Stability Proof

**Proof.**  Combining Equations (21) and (23), from Equation (16) we can obtain

(25) $$\begin{array}{cc} \dot{E} & \leq -\lambda \left(x_{1}^{2}+z^{2}\right)+M{\left(x_{1}^{2}+z^{2}\right)}^{3/2}\\ \; & =-\left(x_{1}^{2}+z^{2}\right)\left[\lambda -M{\left(x_{1}^{2}+z^{2}\right)}^{1/2}\right] \end{array}$$

Let $r=\sqrt{x_{1}^{2}+z^{2}}$, then ${\left(x_{1}^{2}+z^{2}\right)}^{1/2}=r$, and Equation (24) can be written as

(26) $$\dot{E}\leq -r^{2}\left(\lambda -Mr\right)$$

Obviously, when r<λ/M, we have λ−Mr>0, and thus $\dot{E}<0$ (except at the origin). Therefore, we can select

(27) $$\delta =\frac{\lambda }{2M}\;(\mathrm{or}\;\mathrm{any}\;\mathrm{positive}\;\mathrm{number}\;\mathrm{smaller}\;\mathrm{than}\;\lambda /M)$$

Define the neighborhood of the origin

(28) $$U=\left\{\left(x_{1},z\right)|x_{1}^{2}+z^{2}<\delta^{2}\right\}$$

Then for any $\left(x_{1},z\right)\in U\setminus \left\{\left(0,0\right)\right\}$, we have $\dot{E}<0$.

Comprehensive analysis:

1. *E* is positive definite in *U* (E(0,0)=0, and E>0 when $\left(x_{1},z\right)\neq \left(0,0\right)$).
2. $\dot{E}$ is negative definite in *U* (strictly less than 0 except at the origin).

□

According to the Lyapunov stability theorem, under the parameter condition c>γ/4, the equilibrium point (0,0) of the sliding mode system (9) is locally asymptotically stable. This means that as long as the initial state $\left(x_{1}\left(0\right),z\left(0\right)\right)$ is sufficiently close to the origin, the system states will converge to the origin over time, which also reveals the energy exchange mechanism between the virtual state and the real state.

The linearization analysis ensures the “unconditional” local exponential stability (which holds for any γ), while the Lyapunov analysis gives a specific estimate of the domain of attraction and reveals the process of energy dissipation. Notably, the finding that γ does not affect local stability has important engineering implications: the coupling strength γ can be adjusted as an independent free parameter to optimize the transient response of the system (such as convergence speed, overshoot suppression, energy consumption, etc.), without compromising the local stability properties of the system. The condition c>γ/4 provides a sufficient criterion for ensuring a non-vanishing domain of attraction.

**Remark 3**  (Conservatism of the DOA estimate)**.** *For the nominal simulation parameters (c=3, γ=2, ρ=2), the eigenvalues of A yield λ≈1.382, M=5.0, and thus δ≈0.138. This estimate is conservative due to the norm bounds used in (23). As demonstrated in Section 5.1, convergence is observed from initial states far beyond this bound (e.g., $x_{1}\left(0\right)=10$), indicating that the actual domain of attraction is substantially larger—the empirical convergence region is over 70 times larger than the theoretical estimate. The estimate should be interpreted as a guaranteed—not maximal—stability region.*

**Remark 4**  (Extension to higher-order systems)**.** *For an n-th-order integrator chain, the proposed method can be extended by introducing n−1 virtual states with cascaded product coupling: ${\dot{z}}_{i}=x_{i}x_{i+1}-\rho_{i}z_{i}$ (i=1,…,n−1), with sliding surface $\sigma =x_{n}+\sum_{i=1}^{n-1}c_{i}x_{i}+\sum_{i=1}^{n-1}\gamma_{i}z_{i}$. The Lyapunov analysis extends via block-diagonal quadratic forms, with coupling conditions generalizing c>γ/4. A formal proof is deferred to future work.*

## 5. Simulation Validation

**Figure 1:**
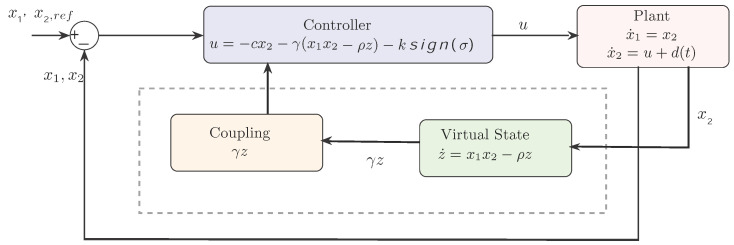
Architecture diagram of virtual state coupled sliding mode controller.

The block diagram of the control system architecture is shown in Figure 1, where γ denotes the coupling strength, ρ the dissipation coefficient, *c* the sliding surface coefficient, *k* the switching gain, δ the boundary layer thickness, and *z* the virtual state.

Simulations were conducted in MATLAB (R2024b) using the fourth-order Runge–Kutta method for numerical integration, with a step size of 0.001 s. The controller parameters are uniformly set as follows: the sliding surface coefficient c=3, the virtual state dissipation coefficient ρ=2, the switching gain k=5, and the boundary layer thickness δ=0.01. The coupling strength γ is set to 0 for the conventional method and to 2 for the proposed method. No actuator amplitude saturation is imposed in the baseline simulations; therefore, the plotted u(t) represents the unconstrained controller output, while sat(σ/δ) only denotes the boundary-layer approximation of the switching function. The physical quantities used throughout this paper, together with their units, are listed in Table 1. The following indicators are adopted to quantify the system performance: maximum deviation $M_{d}=\max\left|x_{1}\right|$, control energy $E_{u}=\int u^{2}dt$, settling time $t_{s}$, number of oscillations $N_{z}$, steady-state error $e_{ss}$, and total variation of the control input $\mathrm{TV}\left(u\right)=\sum |u\left(t_{i+1}\right)-u\left(t_{i}\right)|$ as a chattering indicator.

### 5.1. Validation Under Extended Initial Conditions

To comprehensively examine the robustness of the proposed method against variations in the initial state, with the coupling strength fixed at γ=2, eight representative initial states are selected for the simulation: A. [1,0,0], B. [2,0,0], C. [1,1,0], D. [−1,0,0], E. [5,0,0], F. [10,0,0], G. [1,0,−5], H. [1,0,10]. Among them, Cases A–D correspond to the original four initial conditions; Cases E and F examine large initial position errors far beyond the theoretical DOA estimate (δ≈0.138); Cases G and H verify the robustness to z(0) mismatch.

**Figure 2:**
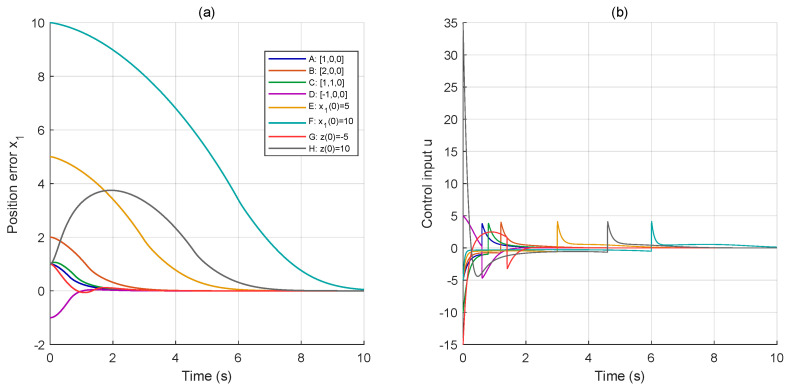
Responseunder extended initial conditions: (**a**) position error; (**b**) control signal.

Figure 2 presents the response curves of the position error $x_{1}\left(t\right)$ and the corresponding control signals u(t) under the eight initial conditions. All trajectories converge smoothly to the origin without overshoot, including Cases G and H, where z(0) deviates significantly from $x_{1}\left(0\right)$. This confirms that stability is preserved even when the initial virtual state is mismatched, and that the empirical domain of attraction is substantially larger than the conservative Lyapunov estimate—convergence is observed from $x_{1}\left(0\right)=10$, approximately 70 times further than the theoretical bound δ≈0.138. The control signal peaks under large initial errors (Cases E and F) reflect the transient control effort required to drive distant states toward equilibrium. Case C (non-zero initial velocity) is nearly coincident with Case A, indicating insensitivity to initial velocity disturbances. Case D is symmetric to Case A, confirming the symmetry of the system dynamics.

### 5.2. Disturbance-Free γ-Tuning Performance

**Figure 3:**
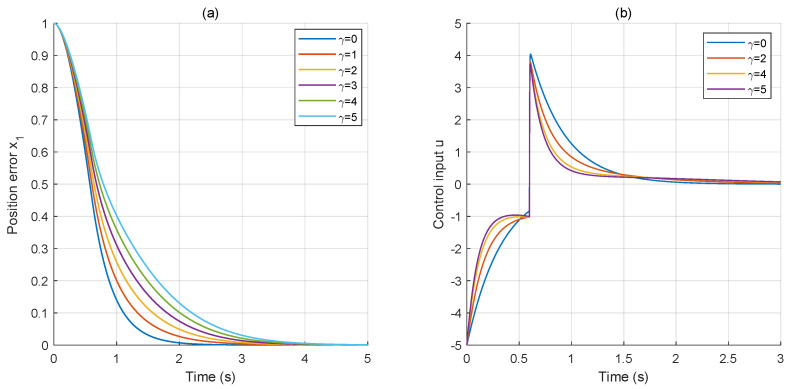
Disturbance-freeresponses for different coupling strengths: (**a**) position error; (**b**) control signal.

To evaluate the influence of the coupling strength γ on the transient performance of the system, the simulation is first conducted under disturbance-free conditions. The initial state is set as $x_{1}\left(0\right)=1$, $x_{2}\left(0\right)=0$, z(0)=0, and the simulation duration is t=5 s. Figure 3 presents the response curves of the position error $x_{1}\left(t\right)$ and the control signal u(t) when γ takes the values of 0, 1, 2, 3, 4, and 5, respectively.

All curves converge smoothly to zero without overshoot. As γ increases, the convergence process gradually slows down: the response is the fastest when γ=0, whereas for γ=5 it requires a noticeably longer time to converge. This trend intuitively reflects the buffering effect of the virtual state on energy flow. A stronger coupling enables more extensive energy exchange between the real state and the virtual state, thereby slowing down the transient process and making the response curve smoother. The control signal amplitude decreases with increasing γ, consistent with the energy buffering interpretation.

**Figure 4:**
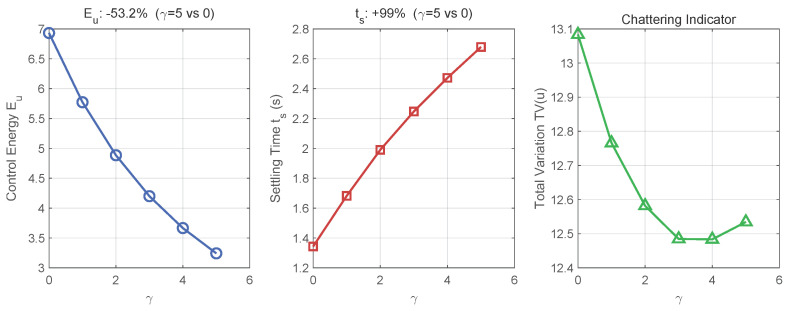
Variationsin control energy $E_{u}$, settling time $t_{s}$, and chattering indicator TV(*u*) with different coupling strengths.

Figure 4 presents the comparison of the control energy $E_{u}=\int u^{2}dt$, the settling time $t_{s}$ (the time to enter the ±0.05 error band), and the chattering indicator TV(*u*) with γ. The control energy decreases monotonically as γ increases: when γ=0, the control energy is 6.93; it drops to 5.77 when γ=1; it is 4.88 when γ=2; it is 4.20 when γ=3; it is 3.67 when γ=4; and it further drops to 3.24 when γ=5. Compared with γ=0, the control energy at γ=5 is reduced by 53.2%. This significant improvement is attributed to the introduction of the virtual state: in conventional sliding mode control (γ=0), the controller must directly provide all the energy to drive the state convergence, whereas after the introduction of the virtual state, part of the energy is temporarily stored in the virtual state and released slowly in the subsequent process, thereby lowering the instantaneous power demand and the total energy expenditure of the entire control process. This energy buffering and redistribution mechanism is a direct embodiment of the core idea of the tennis racket effect in control design. However, improved energy efficiency is achieved at the cost of longer settling time. The settling time increases monotonically with γ: it is 1.34 s when γ=0, 1.68 s when γ=1, 1.99 s when γ=2, 2.25 s when γ=3, and 2.47 s when γ=4, and it increases to 2.68 s when γ=5. From γ=0 to γ=5, the settling time increases by approximately 99%. This trade-off is an inevitable result of the energy buffering mechanism. The chattering indicator TV(*u*) remains within a narrow band (12.48–13.08, relative variation <5%), confirming that the introduction of the virtual state does not significantly increase chattering. In practical applications, the appropriate γ can be selected according to specific performance requirements: if a fast response is required, a smaller γ can be chosen; if energy efficiency is a priority, a larger γ can be selected.

### 5.3. Performance Analysis Under High-Frequency Disturbances

This section focuses on investigating the performance of the proposed method under persistent high-frequency disturbances. A high-frequency disturbance d(t)=100sin(20πt) (unit: m/s^2^) is applied from t=0. By adjusting the coupling strength γ and the dissipation coefficient ρ, the influence of the virtual state on the response smoothness and control energy of the system is systematically evaluated, and the adjustable trade-off relationship between energy and performance is revealed.

**Figure 5:**
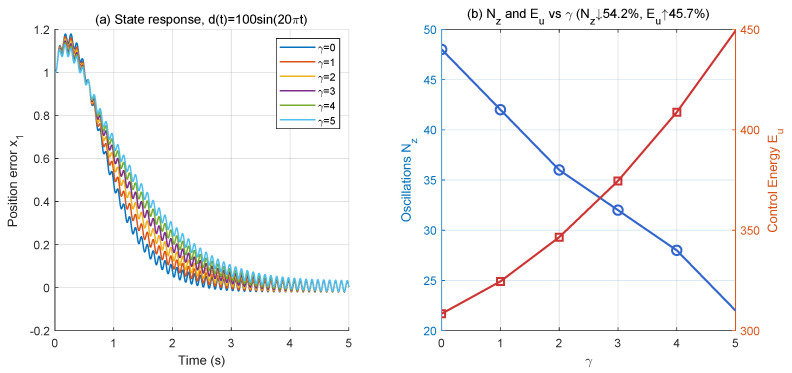
High-frequencydisturbance performance: (**a**) state response; (**b**) $N_{z}$ and $E_{u}$ vs. γ. The arrows indicate the monotonic trends of $N_{z}$ (decreasing) and $E_{u}$ (increasing) as γ increases.

In Figure 5, two curves with opposite trends can be clearly observed: as γ increases from 0 to 5, the number of oscillations $N_{z}$ decreases monotonically (response becomes smoother), while the control energy $E_{u}$ increases monotonically. Specifically, when γ=0 (conventional sliding mode), $N_{z}$ is 48 and $E_{u}$ is 308.4; when γ=2, $N_{z}$ drops to 36 (a reduction of 25.0%), and $E_{u}$ rises to 346.5 (an increase of 12.4%); when γ=5, $N_{z}$ further drops to 22 (a reduction of 54.2% compared with γ=0), and $E_{u}$ rises to 449.3 (an increase of 45.7% compared with γ=0). This result quantitatively reveals the core role of the virtual state under high-frequency disturbances: increasing the coupling strength γ can effectively suppress the oscillations of the system, making the response smoother, but at the cost of increased control energy. This is because maintaining the virtual state dynamics and compensating for its dissipation under strong disturbances requires the controller to supply additional energy.

**Figure 6:**
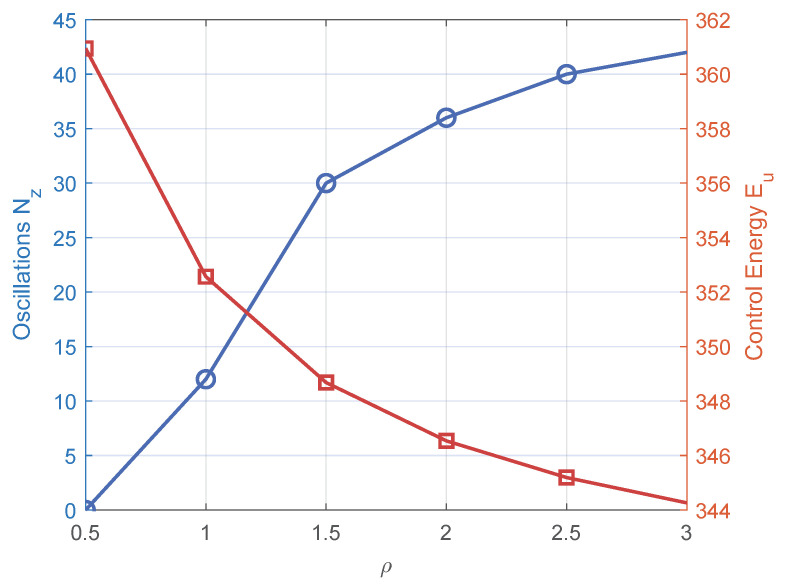
High-frequency disturbance: robustness of $N_{z}$ and $E_{u}$ to ρ (γ=2).

The dissipation coefficient ρ in the dynamics of the virtual state regulates the energy dissipation rate. Figure 6 shows that when ρ=0.5, the dissipation process is slow, and oscillations are completely eliminated ($N_{z}=0$), but $E_{u}$ is as high as 360.9; as ρ increases to 1.5, $N_{z}$ rises to 30, and $E_{u}$ drops to 348.7; when ρ is around 2.0, $N_{z}$ is approximately 36, $E_{u}$ is approximately 346.5, and both tend to be stable; if ρ continues to increase to 3.0, $N_{z}$ slowly rises to 42, and $E_{u}$ slightly drops to 344.3. Overall, in the interval of ρ∈[1.5,3.0], the variation in $N_{z}$ is less than 4% and the variation in $E_{u}$ is less than 0.5%, indicating good robustness to this parameter. In practical engineering, selecting ρ≈c (e.g., ρ=2∼3) achieves a good balance between smooth response and energy efficiency and is insensitive to parameter perturbations.

### 5.4. Comparative Analysis with Benchmark Methods

To benchmark the proposed method, three comparative controllers are implemented under disturbance-free conditions with $x_{1}\left(0\right)=1$: (1) conventional SMC (γ=0) [41], (2) terminal SMC [15] with sliding surface $\sigma =x_{2}+\beta {\left|x_{1}\right|}^{q/p}\;\mathrm{sign}\left(x_{1}\right)$ (β=3, q/p=0.6), and (3) adaptive integral SMC [17] with $\sigma =x_{2}+c_{1}x_{1}+c_{2}\int x_{1}dt$ and adaptive gain update.

**Figure 7:**
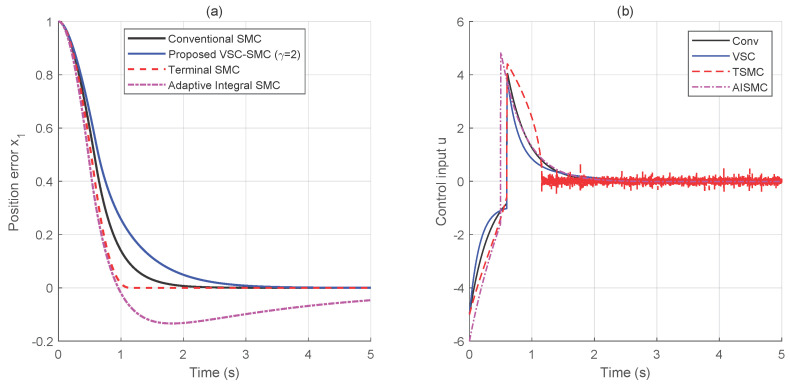
Comparativeanalysis: (**a**) state response; (**b**) control signal.

Figure 7 and Table 2 present the comparative results. The proposed VSC-SMC (γ=2) achieves the lowest control energy among all methods (29.6% lower than conventional SMC, 55.9% lower than terminal SMC, and 58.4% lower than adaptive integral SMC), at the cost of moderately longer settling time compared with conventional and terminal SMC. Terminal SMC achieves the fastest convergence ($t_{s}=0.91$ s) but exhibits a substantially higher chattering level (TV = 519.11) due to its nonlinear sliding surface structure. Adaptive integral SMC achieves a moderate TV (15.69) but at the cost of the longest settling time (4.81 s) and highest energy consumption (11.74), likely due to integral windup effects. Overall, the proposed method provides the best balance among energy efficiency, chattering suppression, and convergence smoothness.

### 5.5. Monte Carlo Robustness Under Parameter Perturbations

To statistically evaluate robustness under simultaneous multi-parameter uncertainties, Monte Carlo simulations (500 trials) are conducted with ±30% random perturbations in all four parameters (c,γ,ρ,k) and ±10% perturbation in initial conditions.

**Figure 8:**
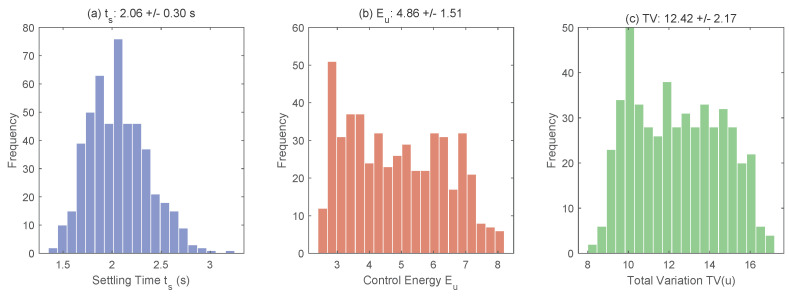
MonteCarlo results (500 trials, ±30% perturbation): histograms of (**a**) settling time, (**b**) control energy, (**c**) TV(*u*).

Figure 8 presents the histograms. All 500 trials converge to the origin, achieving 100% convergence. The settling time has a mean of 2.06 s with a standard deviation of 0.30 s (CV = 14.7%), the control energy has a mean of 4.86 with a standard deviation of 1.51 (CV = 31.1%), and the chattering indicator TV(*u*) has a mean of 12.42 with a standard deviation of 2.17 (CV = 17.4%). No divergent samples are observed. These results confirm that the proposed controller maintains robust and consistent performance under substantial parameter uncertainties.

**Figure 9:**
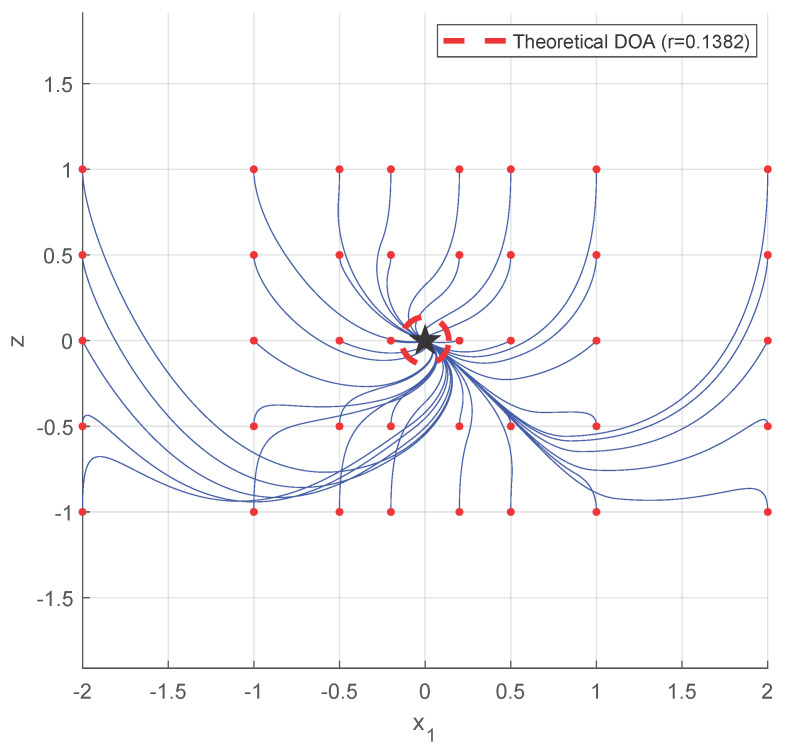
Phaseportrait in the $\left(x_{1},z\right)$ plane with theoretical DOA estimate (dashed circle, r≈0.138) and trajectories from various initial points. The five-pointed star marks the equilibrium point (0,0).

Figure 9 shows the phase portrait of the reduced-order system (9) in the $\left(x_{1},z\right)$ plane. Trajectories from initial points within the grid $x_{1}\left(0\right)\in \left[-2,2\right]$, z(0)∈[−1,1] are plotted. All trajectories converge to the origin, with the theoretical DOA boundary (r≈0.138) shown as a dashed circle. The empirical convergence region is substantially larger than the theoretical guarantee, consistent with Remark 2.

The energy-saving capability of the proposed method (up to 53.2% reduction in $E_{u}$) is comparable to the optimal sliding mode design in [39], while offering the advantage of an independently tunable trade-off via a single parameter γ without requiring online optimization. The disturbance rejection performance is further supported by the piecewise sliding mode approach in [40].

## 6. Conclusions

Inspired by the energy exchange mechanism inherent in the tennis racket theorem, this paper has proposed a novel sliding mode control methodology based on virtual state coupling. By introducing a dynamic virtual state and constructing a sliding surface that incorporates a product coupling term, a coupled dynamical system between the actual system states and the virtual state is established. This formulation enables the active regulation and redistribution of energy across different state channels during the convergence process. Theoretical analysis demonstrates that the origin of the sliding mode system constitutes an exponentially stable equilibrium, and that the coupling strength γ is decoupled from the linearized local eigenvalues while influencing the domain of attraction through c>γ/4. Simulation results corroborate the effectiveness of the proposed approach. Under disturbance-free conditions, the proposed method achieves a substantial reduction in control energy expenditure of up to 53.2% compared with conventional sliding mode control. Under persistent high-frequency external disturbances, the method enhances response smoothness by 54.2% at a controllable energy cost of 45.7%, while exhibiting strong robustness against parametric perturbations. Comparative benchmarks against terminal SMC and adaptive integral SMC confirm that the proposed method achieves the lowest control energy with comparable chattering levels. Monte Carlo simulations (500 trials, ±30% parameter perturbations) demonstrate 100% convergence under simultaneous multi-parameter uncertainties. The proposed method provides an independently adjustable energy–performance trade-off mechanism, suitable for engineering applications in which both transient response quality and energy efficiency are critical.

## Figures and Tables

**Table 1:** Symbols and units of physical quantities.

Symbol	Physical Meaning	Unit
$x_{1}$	Position error	m or rad
$x_{2}$	Velocity	m/s or rad/s
*z*	Virtual state	Normalized *
*c*	Sliding surface coefficient	s^−1^
γ	Coupling strength	Normalized *
ρ	Dissipation coefficient	s^−1^
*k*	Switching gain	m/s^2^
δ	Boundary layer thickness	Same unit as σ
d(t)	External disturbance	m/s^2^
$M_{d}$	Maximum deviation	m
$E_{u}$	Control energy	(m/s^2^)^2^·s
$t_{s}$	Settling time	s
$N_{z}$	Number of oscillations	Dimensionless
$e_{ss}$	Steady-state error	m
TV(u)	Total variation of control	m/s^2^

* Note: *z* and γ are treated as normalized quantities in the simulations.

**Table 2:** Performance comparison under disturbance-free conditions.

Method	$E_{u}$	$t_{s}$ (s)	TV(*u*)	Overshoot
Conventional SMC [41]	6.93	1.34	13.08	0%
Terminal SMC [15]	11.07	0.91	519.11	0%
Adaptive Integral SMC [17]	11.74	4.81	15.69	0%
Proposed VSC-SMC (γ=2)	4.88	1.99	12.58	0%

## Data Availability

The original contributions presented in this study are included in the article. Further inquiries can be directed to the corresponding author.
